# Prostaglandins in Marine Organisms: A Review

**DOI:** 10.3390/md17070428

**Published:** 2019-07-23

**Authors:** Federica Di Costanzo, Valeria Di Dato, Adrianna Ianora, Giovanna Romano

**Affiliations:** Marine Biotechnology Department, Stazione Zoologica Anton Dohrn Napoli, Villa Comunale, 80121 Napoli, Italy

**Keywords:** prostaglandins, clavulones, punaglandins, thromboxane, inflammation, marine vertebrates, marine invertebrates, diatoms, macroalgae

## Abstract

Prostaglandins (PGs) are lipid mediators belonging to the eicosanoid family. PGs were first discovered in mammals where they are key players in a great variety of physiological and pathological processes, for instance muscle and blood vessel tone regulation, inflammation, signaling, hemostasis, reproduction, and sleep-wake regulation. These molecules have successively been discovered in lower organisms, including marine invertebrates in which they play similar roles to those in mammals, being involved in the control of oogenesis and spermatogenesis, ion transport, and defense. Prostaglandins have also been found in some marine macroalgae of the genera *Gracilaria* and *Laminaria* and very recently the PGs pathway has been identified for the first time in some species of marine microalgae. In this review we report on the occurrence of prostaglandins in the marine environment and discuss the anti-inflammatory role of these molecules.

## 1. Introduction

Marine organisms have a great potential to produce a vast variety of bioactive molecules with high antibiotic, anti-proliferative, and anti-inflammatory activity [1]. The biodiversity hosted by the oceans is greater than in terrestrial environments [2] but nonetheless, marine bioresources are still underexplored, and many species await to be discovered. 

The high probability to find new interesting bioactive molecules from marine organisms has fostered the effort of the scientific community to adopt new technologies and approaches to increase the success of biodiscovery from marine resources, with a main focus on products with antibiotic, antitumor and anti-inflammatory activities. Of particular interest is the search for new anti-inflammatory compounds since inflammation processes are often related to the onset of chronic pathologies and tumors.

Indeed, inflammation processes represent a fundamental way to restore the original equilibrium of a cell or tissue whose physiology has been impaired by damaging stimuli [3]. At the same time, if inflammation is not blocked it can stimulate a cascade of events that eventually lead to serious diseases such as cancer and autoimmune disorders [4]. The inflammation-resolution process can have different features depending on the type of tissue and injurious stimulus [5], therefore, specific types of pro-resolution stimuli or drugs may be necessary [6]. Both the onset and the resolution of the inflammation are active processes that involve a complex interplay of different molecules [7] like chemokines, cell adhesion molecules, proteolytic enzymes, eicosanoids [8], reactive oxygen species (ROS), and reactive nitrogen species (RNS) [9,10]. Among these, eicosanoids deriving from oxidation of polyunsaturated fatty acids (PUFA) through cyclooxygenase (COX) and lipoxygenase (LOX) pathways play a pivotal role both in the onset and in the resolution of inflammation [9]. The main products of COX enzymes are prostaglandins (PGs), fatty acid derivatives with a molecular structure based on 20 carbon atoms that share a prostanoic acid skeleton. 

Prostaglandin E_2_ was the first PGs to be identified in the early 1930s in human seminal plasma by Von Euler [11] and, independently, by Goldblatt [12] although the chemical structures were determined only 30 years later by Bergström, Samuelsson, and co-workers [13]. 

PGs are very well studied and described in mammals where they are active in a great variety of physiological and pathological processes such as smooth muscle and vaso-tone regulation, signaling, hemostasis sleep-wake regulation, reproduction, and especially inflammation. In these organisms, they are synthetized and released in response to external stimuli [14] and rapidly inactivated by metabolizing enzymes after they have accomplished their function [15]. 

PGs represent very important and interesting lipid mediators in all vertebrates and in both terrestrial and marine invertebrates [16,17]. Plants utilize chemically related molecules (jasmonic acid) that have a defensive role, similarly to PGs [16,18,19]. Nevertheless, in some plant species like onions and poplar, a few PGs have also been identified [20].

In the marine environment, prostaglandins have been reported both in vertebrates such as carps, sheatfish and leopard sharks, and invertebrates such as sea squirts, mussels, scallops, crawfish, blue crabs, and sea-anemones and in some macroalgae as the red alga *Gracilaria asiatica* C. F. Zhang & B. M. Xia, 1985 and the brown alga *Laminaria digitata* (Hudson) J. V. Lamouroux, 1813. 

The earliest report about the presence of prostaglandins in marine invertebrates was by Weinheimer and Spraggins (1969) that discovered 15-epi-prostaglandin A_2_ and its acetate methyl ester in the gorgonian-type coral *Plexaura homomalla* Esper, 1794 [21]. The discovery of high PGs levels in gorgonians contributed to the rapid growth of the study and application of PGs in the pharmaceutical and biomedical sector [22].

Very recently, Di Dato et al. (2017) described the presence of all the three series of PGs molecules also in diatoms, an ecologically successful group of marine microalgae [23]. 

In this review, we present a short background on prostaglandin structure and function and give an updated overview of the presence of PGs in marine organisms, discussing the anti-inflammatory role of PGs from the marine environment.

## 2. Structure, Biosynthesis, and Activity of Prostaglandins in Mammals

**Figure 1:**
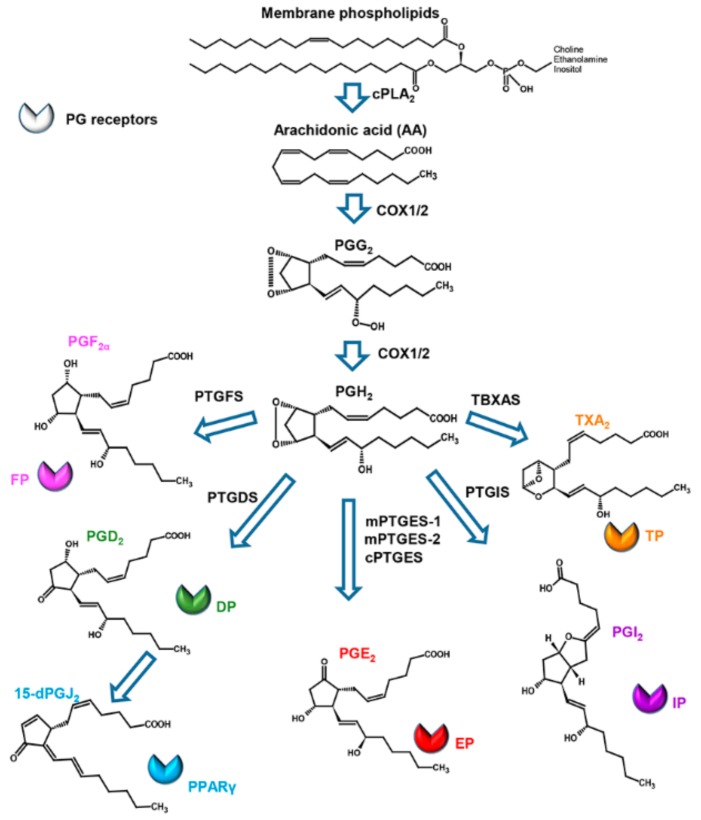
Prostaglandin biosynthetic pathway. Enzymes involved in the pathway are reported next to the arrows. For the abbreviation, refer to the text. Modified from [24].

Prostaglandins consist of a cyclopentanone nucleus with two side chains. Primary prostaglandins, which include prostaglandin D_2_ (PGD_2_), prostaglandin E_2_ (PGE_2_), prostaglandin F_2α_ (PGF_2α_), and prostaglandin I_2_ (PGI_2_), contain a 15-hydroxyl group with a 13,14-trans double bond (Figure 1).

Currently, three classes of prostaglandins are categorized, based on the number of double bonds present within the molecule and on the fatty acid precursor. Prostaglandins belonging to the series 1 have one double bond and derive from 8,11,14-eicosatrienoic acid (di-homolinolenic acid, ETrA), those of the series 2 have two double bonds and derive from 5,8,11,14-eicosatetraenoic acid (arachidonic acid, ARA), and those of the series 3 have three double bonds and derive from 5,8,11,14,17-eicosapentaenoic acid (EPA).

Their nomenclature comprises 10 specific molecular groups, identified by the letters A through J, that differ by variation in the functional groups attached to positions 9 and 11 of the cyclopentane ring. For PGF, the additional subscript “α” or “β” denotes the spatial configuration of the carbon 9 hydroxyl group. For additional details on the nomenclature of prostaglandins see Lands, 1979 [25].

PGs action is mediated by the interaction with specific receptors present on the plasma membrane. These are transmembrane G-protein coupled receptors (GPCR), named as prostaglandin EP receptor (EP), prostaglandin F_2α_ receptor (FP), prostaglandin DP receptor (DP), and prostacyclin I_2_ receptor (IP) receptors, that are highly selective for PGE_2_ PGF_2α_, PGD_2_, and PGI_2_, respectively [26]. The EP family comprises four isoforms (EP_1-4_) that play a relevant role in inflammation processes [27]. The downstream signaling of this receptor family is responsible for the pleiotropic ability of PGE_2_ to activate different processes, including cell proliferation, apoptosis, angiogenesis, inflammation, and immune surveillance in different cell types [24].

Most prostaglandins display a marked structure-activity specificity mainly determined by substitutions in the cyclopentanone ring and the degree of unsaturation of the side chains. They exert their function once secreted into the extracellular medium, where they are rapidly metabolized by 15-hydroxyprostaglandin dehydrogenase (15-OH-PGDH). This enzyme selectively oxidizes the hydroxyl group at carbon 15 into a 15-keto derivative [28] accompanied by a substantial loss of biological activity. 

Prostaglandins derive from the sequential actions of highly specific enzymes (Figure 1). Their synthesis is initiated by phospholipases A_2_ (PLA_2_), a family of enzymes that hydrolyze membrane phospholipids at the sn-2 position, liberating free fatty acid precursors, mainly ARA [15]. These enzymes represent a key step in the PG biosynthetic pathway, being regulated by Ca^2+^ binding and phosphorylation by mitogen-activated protein kinase (MAPK) in response to different stimuli. Membrane-released ARA is then rapidly converted through the cyclization and inclusion of molecular oxygen in the precursor by the action of cyclooxygenase (COXs) enzymes into the unstable metabolite PGG_2_, which is subsequently reduced to PGH_2_ by the same enzyme [14]. Cyclooxygenases exist in a substrate-limiting environment; thus, liberation of fatty acids from esterified stores results in the prompt formation of the products. There are two major COX isoforms; COX-1 is constitutively active and present in most cells in the body; expression of the COX-2 isoform is inducible in many tissues by pro-inflammatory and mitogenic stimuli, such as cytokines [29]. The specific transformation of the first product PGH_2_ to other PGs and thromboxanes (TXs) by downstream enzymes is complexly orchestrated and is cell specific, since each cell tends to form mainly one of these compounds as the major product. For example, in brain and mast cells, PGH_2_ is converted to PGD_2_, whereas it is converted in PGF_2α_ in the uterus; from the same precursor, vascular endothelial cells produce PGI_2_ (prostacyclin) and platelets release thromboxane A_2_ (TXA_2_). 

The conversion to PGE_2_, the most widespread PG, is due to PGE synthase-1, also present in different isoforms in mammals: microsomal PGE synthase-1 (mPTGES-1), mPTGES-2, and cytosolic PGE synthase (cPTGES), the latter of particular interest since frequently associated to the tumorigenic activity of PGE_2_ [30]. 

**Figure 2:**
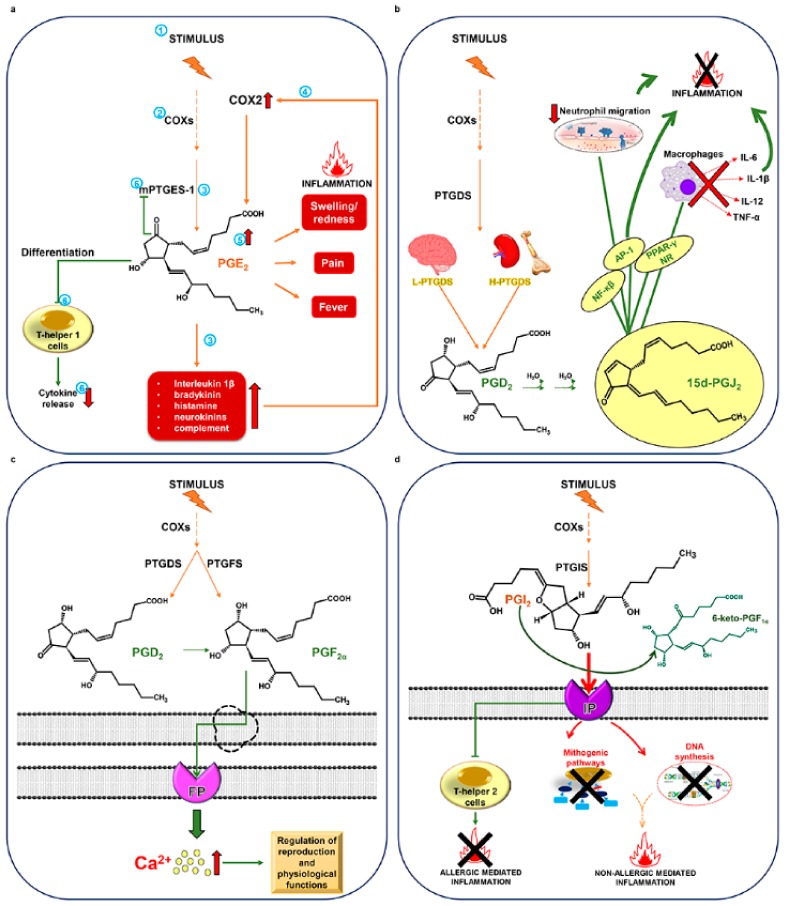
Prostaglandins and inflammation. (**a**) Prostaglandin E_2_ (PGE_2_) stimulation of inflammatory response. Numbers indicate the sequence of events from PGE_2_ synthesis to stimulation of inflammation trough positive feedback on cyclooxygenase (COX)-2 and negative feedback on microsomal PGE synthase-1 (mPTGES-1); (**b**) The anti-inflammatory role of prostaglandin J_2_ (PGJ_2_); (**c**) prostaglandin F_2α_ (PGF_2α_) signaling; (**d**) prostaglandin I_2_ (PGI_2_) signaling. For details, refer to the text.

Prostaglandin E_2_ synthesis is a key event for the development of the three principal signs of inflammation: swelling, redness, pain and fever. Moreover, PGE_2_ also contributes to the amplification of the inflammatory response by enhancing and prolonging signals produced by pro-inflammatory agents, such as interleukin 1α bradykinin, histamine, neurokinins, and complement [31,32]. These signals, in turn, can increase COX-2 expression thus further increasing PGE_2_ synthesis. This PG, however, can have a double, inverse role, being also able to act as an immunosuppressant, repressing the differentiation of T helper 1 cells and limiting cytokine release and further prostaglandin synthesis by activating a negative feedback on mPGES-1 (Figure 2a). 

PGD_2_-synthesizing enzymes exist as two distinct genes coding for hematopoietic- and lipocalin-type PGD synthases (H-PTGDS and L-PTGDS, respectively). H-PTGDS is generally localized in the cytosol of immune and inflammatory cells, whereas L-PTGDS has a tissue-based expression [33]. The activity of PGD_2_ has been mainly associated with inflammatory conditions, being involved in immunologically relevant functions. Its action seems to be mediated by the non-enzymatic production of the PGJ_2_ family, which occurs through a spontaneous dehydration in aqueous solutions. One of the most studied PGJ_2_ metabolites, 15-deoxy-Δ12-PGJ_2_ (15dPGJ_2_), showed anti-inflammatory properties based on interaction with the intracellular targets Nuclear Factor kappa-light-chain-enhancer of activated B cells (NF-κB), Activator Protein 1 (AP-1), and Peroxisome Proliferator-Activated Receptor gamma (PPAR-γ) [31]. 15dPGJ_2_ also induces the reduction of neutrophil migration and inhibits the release of Interleukin 6 (IL-6), Interleukin 1β (IL-1β), Interleukin 12 (IL-12), and Tumor Necrosis Factor-α (TNF-α) from macrophages (Figure 2b).

PGD_2_ can be further metabolized to PGF_2α_, although the latter can also be synthesized from PGH_2_ via PGF synthase. PGF_2α_ acts via FP receptors, resulting in the elevation of intracellular free calcium concentrations that regulates numerous important physiological functions related to reproduction linking multiple molecular mechanisms that are fine-tuned coordinated events in mammalian physiology [34] (Figure 2c). The emerging role of PGF_2α_ in acute and chronic inflammation has opened new opportunities for the design of novel anti-inflammatory drugs.

A non-enzymatic dehydration reaction is also responsible for the formation of the PGA series from the corresponding PGE. The PG series A and J contain an α,β-unsaturated carbonyl group within the cyclopentenone ring, which seem to contribute to the anti-inflammatory activity of these PGs [35]. 

The enzyme prostaglandin I synthase (PGIS), a member of the cytochrome P450 superfamily, specifically converts PGH_2_ to PGI_2_ [33]. This PG has anti-mitogenic activity and inhibits DNA synthesis through specific IP receptors. It mediates pro-inflammatory stimuli in non-allergic acute inflammation, while acting as an anti-inflammatory mediator in Th2-mediated allergic inflammatory responses. Soon after having exerted its action, PGI_2_ is rapidly converted by non-enzymatic processes to the inactive product 6-keto-PGF_1α_ (Figure 2d).

## 3. Prostaglandins and Derivative Molecules in Marine Organisms

Similar to terrestrial vertebrates, in marine vertebrates prostaglandins are involved in reproduction, osmoregulation, regulation of oxygenation, and cardiovascular system [36]. Marine invertebrates, including sponges, corals, and molluscs, also contain a wide range of prostaglandins, many of which are of the conventional type (PGA_2_, PGE_2_, PGD_2_, PGF_2α_), with similar functions as in mammals (reproduction, ion transport) and are also probably used as defense compounds [17]. Interestingly, in some invertebrates, PGs are able to perform different actions based on the tissue or compartment localization [37]. 

Complex marine photosynthesizing organisms, like some genera in the brown, green, and red algal groups (respectively, *Laminaria*, *Euglena*, and *Gracilaria* species) express the cyclooxygenase gene synthesizing PGE_2_, PGF_2α_, and other PGs whose functions are not yet known, but seem to be associated to a defensive role [17].

### 3.1. Corals

**Figure 3:**
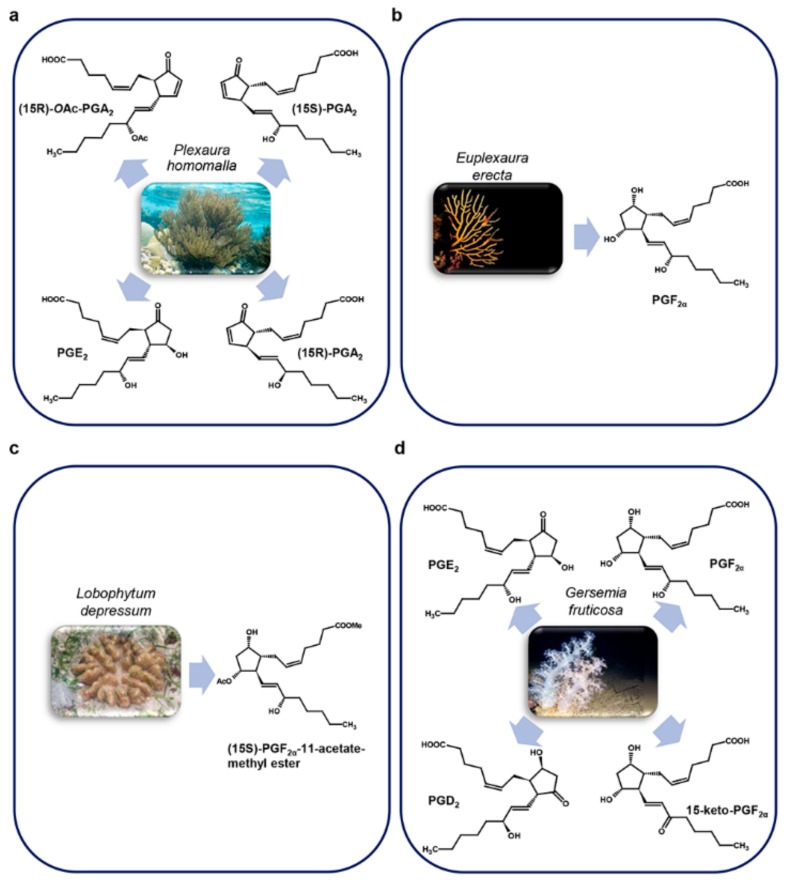
Corals prostaglandins. (**a**) Plexaura homomalla; (**b**) Euplexaura erecta; (**c**) Lobophytum depressum; (**d**) Gersemia fruticosa.

Among marine invertebrates, corals represent a very interesting group producing specific PGs that are not present in mammals. The Caribbean gorgonian *Plexaura homomalla* was indeed the species in which PGs were firstly identified in a marine organism, representing also a major source of these compounds in nature [38] (Figure 3a).

Weinheimer A. and Spraggins R. were the first to perform PGs extraction in *P. homomalla*, identifying in its dry cortex 15-epi-PGA_2_ and its methyl ester acetate (respectively 0.2% and 1.3%) [15] in an R-configuration on their C-15 asymmetric center, a configuration not present in mammals. In addition to (15R)-epi-PGA_2_, also (15R)-PGE_2_, its methyl ester and a complex mixture of other prostaglandins were subsequently found by Light R. and Samuelsson B. [39]. However, these molecules were found also in S configurations [40] and the occurrence of one of the two seems to be related to the geographical distribution of *P. homomalla* [38].

The high PGs concentration in these invertebrates stimulated the attempt to use them as precursors for the chemical synthesis of the biologically active prostaglandins A_2_, E_2_, and F_2α_, since their chemical synthesis in large-scale for medical and pharmaceutical purposes was complicated by the necessity of 16 different chemical reactions [41]. 

The observation that *P. homomalla* present in coral reefs is not commonly eaten by fishes, led Gerhart [42] to hypothesize that the high amount of PGs they contain could be used as chemical defense against potential predators, since it is known that in mammals they can cause vomiting and nausea when administered orally. Indeed, oral doses of both (15R)-PGA_2_ and (15S)-PGA_2_ caused vomiting in a test with fishes, while the PGA_2_ present in the surrounding water did not cause any effect [42]. However, the hypothesized defensive role seems not to be realistic since PGA_2_ is stored in coral tissues only as acetoxy methyl esters, whose conversion to (15R)-PGA_2_ (the compound tested by Gerhart) needs about 24 hours [40]. Moreover, while (15R)-PGA_2_ inhibits fishes from feeding, the acetoxy methyl ester form, orally delivered, does not show any repellent effect. For this reason, as the process of production of the active (15R)-PGA_2_ seems too slow to provide the coral with an effective defense mechanism, the function of these molecules in corals is still an open question.

The R-prostaglandins extracted from *P. homomalla* collected on the Island of San Andreas, in the Caribbean Ocean, were also tested in vivo on mouse ear edema induced by 12-O-tetradecanoylphorbol-13-acetate (TPA) and in vitro, in anti-inflammatory screenings as leucocyte degranulation, myeloperoxidase (MPO), and elastase enzymatic activity inhibition. The results showed that (15R)-PGE_2_ and, to a lesser extent, (15R)-O-AcPGA_2_ had an anti-inflammatory activity in vivo and in vitro (Table 1). In particular, in the leucocyte degranulation assay, (15R)-PGE_2_ greatly inhibited the release of both MPO and elastase, while the other prostaglandins tested were moderately active in the inhibition of elastase release but not of MPO [43].

*Euplexaura erecta* Kükenthal, 1908 [56] (Figure 3b), *Lobophytum depressum* Tixier-Durivault, 1966 [57] (Figure 3c), and *Gersemia fruticosa* Sars, 1860 [58] (Figure 3d) also produce PGs. Interestingly, PGF_2α_ and its derivatives were principally found in these species: PGF_2α_ in *E. erecta* [56]; (15S)-PGF_2α_-11-acetate methyl ester, its 18-acetoxy derivative compound and their corresponding free carboxylic acids in *L. depressum* [57]; PGF_2α_ and 15-keto-PGF_2α_ together with PGD_2_, PGE_2_ in *G. fruticosa* [58]. 

In addition to the identification of PGs molecules in corals, also the enzyme responsible for their synthesis, the cyclooxygenase, was isolated and characterized for the first time from *P. homomalla* and *G. fruticosa* [59].

### 3.2. Other Marine Invertebrates

One of the first studies on marine invertebrates, excluding corals, was a comparative analysis done by Christ E. and Van Dorp D., on representative species in the Mollusca (*Mytilus*), Crustacea (*Homarus*), and Cnidaria (*Cyanea*) phyla versus terrestrial animals [60]. The authors were able to find PGs, particularly PGF_1α_ but only at very low levels. In some cases, the arachidonic acid precursor was also not detectable making it difficult to assert the existence of PGs and their functional role [60]. These results were confirmed in extended studies including more species in the Chordata, Mollusca, Cnidaria, and Crustacea phyla [16,61,62]. 

**Figure 4:**
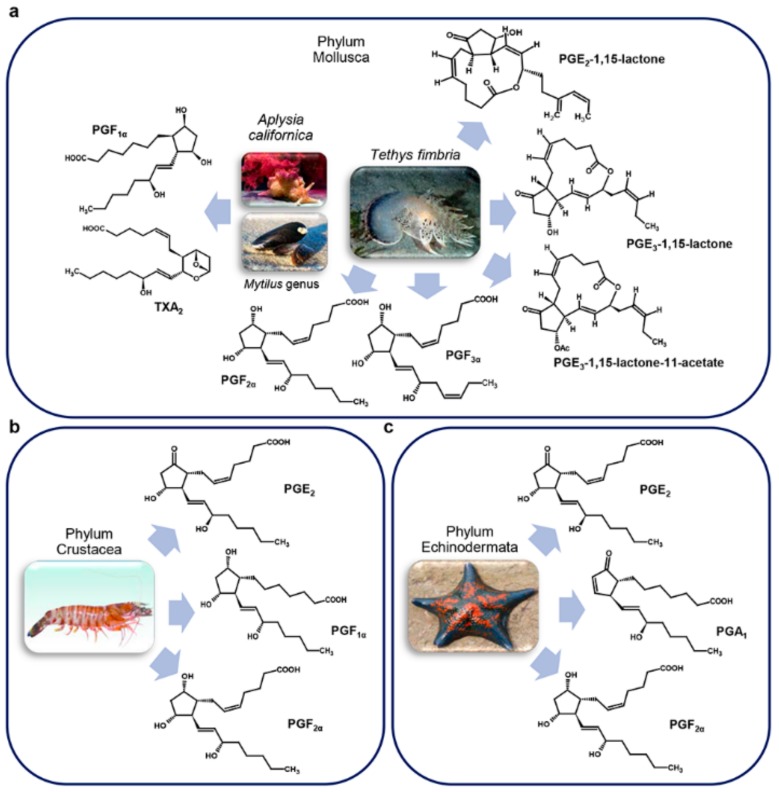
Marine invertebrate prostaglandins. (**a**) Molluscs; (**b**) Crustaceans; (**c**) Echinoderms. Except for *Tethys fimbria* and *Aplysia californica*, pictures show organisms that are only representatives of each phylum. For details, refer to the text.

With the improvement of instrument sensitivity, it was possible, more than 10 years later, to conduct functional studies on PGs in molluscs (Figure 4a) and crustaceans (Figure 4b). 

Results obtained highlighted different roles for PGs in marine invertebrates, like reproduction, regulation of ion flux, and thermoregulation and fever, mediated respectively by PGF_2α_, PGE_2_, and PGE_1_ (Table 2) [63].

In the mollusc *Modiolus demissus* Dillwyn, 1817 the uptake and binding of prostaglandins by gills was investigated, considering that bivalves can both synthetize and accumulate PGs from the surrounding medium [71]. The study revealed the presence of tissue specific, time and pH dependent, PGs binding sites with higher affinity to PGA_2_ with respect to PGE_2_ or PGF_2α_ [71]. 

Moreover, three prostaglandin-lactones (PGE_2_-1,15-lactone, PGE_3_-1,15-lactone, and PGE_3_-1,15-lactone-11-acetate) of the E and F series were identified in the mantle and body, respectively, of the nudibranch mollusc *Tethys fimbria* Linnaeus, 1767 (Figure 4a). High quantities of a complex mixture of PGF_2α_ and PGF_3α_ 1,15-lactones fatty acid esters (PLFE) were found in its eggs, particularly in mature ovotestis but not in immature ones suggesting a role for PLFE in mollusc reproduction [67]. Altogether, these results led to hypothesize a multiple biological role of prostaglandin-lactones, precursors of PGEs, as defense allormones, and involved in the control of smooth muscle contraction, and egg production and fertilization, depending on their body localization (Table 2) [37].

Crustaceans (Figure 4b) also use PGs for physiological functions. Indeed, PGE_2_ and PGF_2α_ were identified in the ovary of the prawn *Marsupenaeus japonicus* Spence Bate, 1888 where they participate in ovarian maturation (Table 2) [64] whereas in the crab *Carcinus maenas* Linnaeus, 1758 they are produced in blood cells following a stimulus induced with a calcium ionophore, in the presence of exogenous fatty acids (FA) [72]. 

Echinoderms (Figure 4c) also represent a source of PGs. Among all the echinoderms analyzed, the starfish *Patinia pectinifera* Muller and Troschel, 1842 had the highest amount of prostaglandins [73]. PGE_2_ and PGF_2α_ have been identified from the sea cucumber *Stichopus japonicus* Selenka, 1867 using TLC [73]. A study conducted with PGs-3H evidenced that the sea urchin *Arbacia punctulata* Lamarck, 1816 could accumulate PGs from the surrounding water into its gut or stomach [74], and to accumulate PGA_1_ both in fertilized and unfertilized eggs, with fertilized eggs accumulating more PGs than unfertilized eggs [74].

*Strongylocentrotus nudus* A. Agassiz, 1864 and *S. intermedius* A. Agassiz, 1864 were also reported to have a PGs-like activity in their inner organs [74]. 

### 3.3. Marine Vertebrates

**Figure 5:**
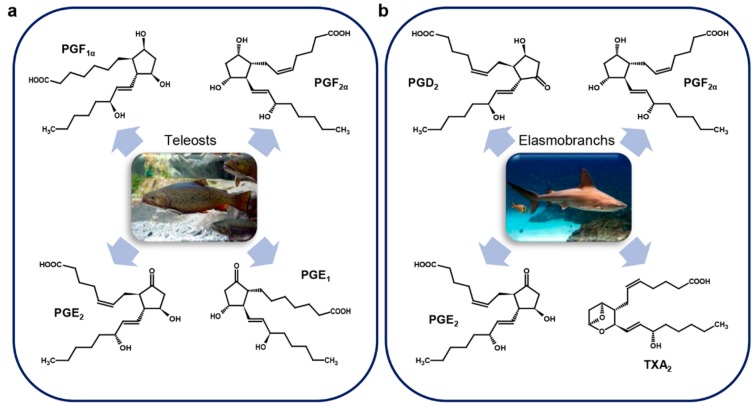
Marine vertebrate prostaglandins. (**a**) Teleosts (Salmo sp., Paralichthys olivaceus, Oncorhynchus keta, Thunnus thynnus, Salvelinus fontinalis, Pleuronectes platessa, Opsanus tau); (**b**) Elasmobranchs (Triakis scyllia, Scyliorhinus canicula). Pictures show organisms that are only representatives of each class. For details, refer to the text.

As already mentioned, in fish PGs are involved in several processes, such as ovulation, spawning, osmoregulation, regulation of branchial ion fluxes and of the cardiovascular system [36]. Christ and Van Dorp in their comparative studies on PGs considered not only invertebrates, but also fish [60]. They identified moderate yields of PGE_1_ in freshwater fish and lower yields in homogenates of gills of *Salmo* sp. [60]. Successively, PGE_2_ was identified for the first time in the testis of the flounder *Paralichthys olivaceus* Temminck & Schlegel, 1846, PGF_1α_ in the semen of the salmon *Oncorhynchus keta* Walbaum, 1792 and PGE_2_ and PGF_2α_ in the testis of the tuna *Thunnus thynnus* Linnaeus, 1758 [65] (Figure 5a). 

In order to explain the reason why prostaglandins are present in these marine animals, although they are oviparous, it was suggested that the identified prostaglandins might be used for the contraction of smooth muscle during ejaculation and for the metabolism of testis in lower animals as in mammals (Table 2) [65]. After this study, PGE_2_ was isolated and subsequently identified in the gastrointestinal tract and in the skin of the shark *Triakis scyllia* Müller & Henle, 1839 [16,75] (Figure 5b).

In the brook trout *Salvelinus fontinalis* PGE_2_ and PGF_2α_ (Figure 5a) are synthetized in the follicle wall of mature oocytes, but the highest quantity of prostaglandins was found in the extra-follicular tissue [76]. PGE_2_ was also found in the skin of *Pleuronectes platessa* Linnaeus, 1758 (Figure 5a) in response to the fungal extract of *Epydermophyton floccosum* (Harz) Langeron & Miloch, 1930 known be an inducer of erythema [77]. 

Leucocytes of the dogfish *Scyliorhinus canicula* Linnaeus, 1758 secerned high levels of PGE_2_, PGD_2_ and PGF_2α_ (Figure 5b) when exposed to the Ca^2+^ ionophore A23187 [78]. PGE_2_ and PGF_2α_ were found in the red blood cells of the toadfish *Opsanus tau* Linnaeus, 1766 [79] (Figure 5a).

### 3.4. Macroalgae

#### 3.4.1. Red Macroalgae

**Figure 6:**
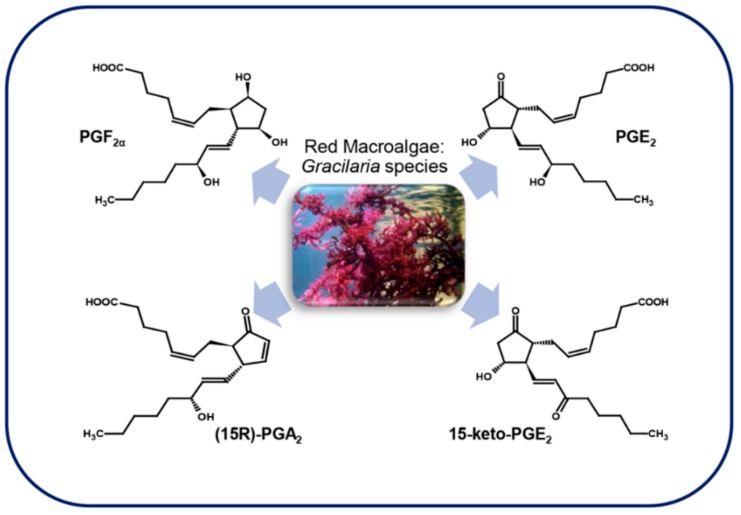
Red algae prostaglandins. The picture shows an organism representative of the genus. For details, refer to the text.

Marine red algae are rich in C20 polyunsaturated fatty acids that, in animals, are precursors of prostaglandins, thromboxane, and other eicosanoids. Although the presence of the prostaglandin-endoperoxide pathway has been demonstrated in non-mammal marine vertebrates and invertebrates, for a long time less was known about these enzymes in non-animal organisms. The first report about the presence of prostaglandins in macroalgae (Figure 6) described the identification of PGE_2_ and PGF_2α_ in *Gracilaria lichenoides* Greville, 1830 [80].

The genus *Gracilaria*, that comprises algae used in the food and cosmetic industry, is very rich in ARA, varying from 45.9% and 62.0% of the total FA depending on the season. Among these, *G. vermiculophylla* (Ohmi) Papenfuss, 1967 is one of the algae with the highest content of ARA [81]. This, with other *Gracilaria* species such as *G. asiatica* and *G. chorda* Holmes, 1896, seem to be responsible for a gastrointestinal disorder known as “onogori” poisoning in Japan when it is eaten raw [18]. The possible reason for this poisoning seems to be the fact that the COX contained in raw seaweeds use the highly unsaturated fatty acids to produce great amounts of PGE_2_ in the stomach of victims in a short lapse of time [81]. *G. vermiculophylla* is a source of different types of prostaglandins besides PGE_2_, such as PGA_2_, PGF_2_, 15-keto-derivatives of prostaglandins, as well as other eicosanoids [82], whereas the congeneric species *G. asiatica* produces only 15-keto-PGE_2_ [83]. *G. vermiculophylla* use PGA_2_, PGE_2_, 15-keto-PGE_2_ and other eicosanoids as wounding-activated chemical defense molecules (Table 2) [66], and recently a COX gene producing PGF_2α_ was cloned from this alga and heterologously expressed [84]. 

Other evidence of prostaglandins as defense molecules in red algae is the fact that gametophytes of the alga *Chondrus crispus* Stackhouse, 1797, when challenged with pathogens, metabolize C20 and C18 PUFAs not only into the corresponding hydroperoxides and derivatives, but also into molecules with mass fragmentation patterns very similar to prostaglandin B_1_ and B_2_ [85]. Furthermore, when the crude extract of this alga was treated with 50–100 µM methyl jasmonate for 6 h, PGA_2_ and 15-keto-PGE_2_ were identified [86]. 

#### 3.4.2. Brown Macroalgae

**Figure 7:**
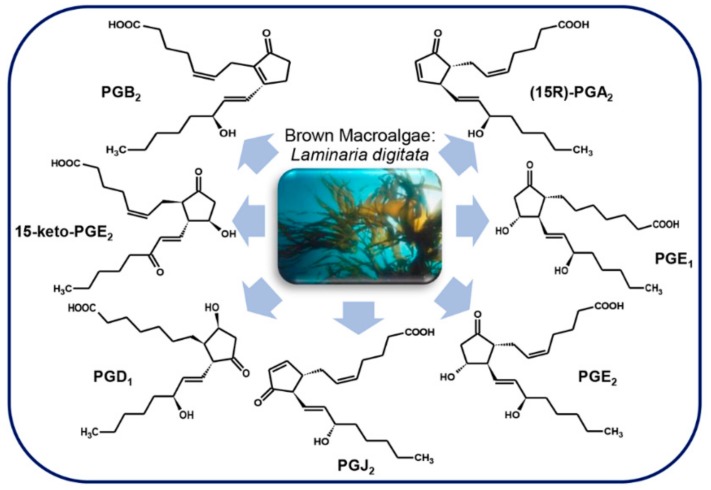
Brown algae prostaglandins: only *Laminaria digitata* has been studied for prostaglandins content. For details, refer to the text.

Much less is known about the presence of prostaglandins in brown algae, and most studies have mainly focused on the brown algal kelp *Laminaria digitata*. Ritter et al. [19] showed that this brown alga uses the generation of oxylipins as a protective mechanism against stress conditions induced by an excess in copper (Table 2). Indeed, high concentrations of copper led to oxygen reactive species (ROS) accumulation in *L. digitata* cells, and consequently to a cascade of cellular responses. One of these responses was a significant release of PUFAs after 24 h of treatment, followed by the generation of oxylipins. Among complex oxylipins, also PGE_1_ and PGD_1_, deriving from ETrA and PGJ_2_, PGA_2_, 15-keto-PGE_2_ and PGB_2_, deriving from ARA were identified for the first time in brown algae [19]. Successively, the same authors showed that PGA_2_ does not simply modulate but also triggers an oxidative response in *L. digitata* that, differently from the response induced by other molecules like methyljasmonate and lipopolysaccharides, occurs in seconds after treatment and in a dose-response-like manner. The authors hypothesized that PGA_2_ can activate the generation of two different sources of ROS that can be used by the brown alga as defense molecules as in other marine organisms [68] (Figure 7). 

### 3.5. Microalgae

PGs were discovered for the first time in diatoms, phytoplanktonic marine microalgae, only in 2017 [23]. Diatoms are a rich source of polyunsaturated fatty acids (PUFA), that, in some species, are precursors of polyunsaturated aldehydes (PUAs), i.e., oxylipins with pro-apoptotic and anti-proliferative activity, with a defensive role against predator (copepods) grazing [87]. 

**Figure 8:**
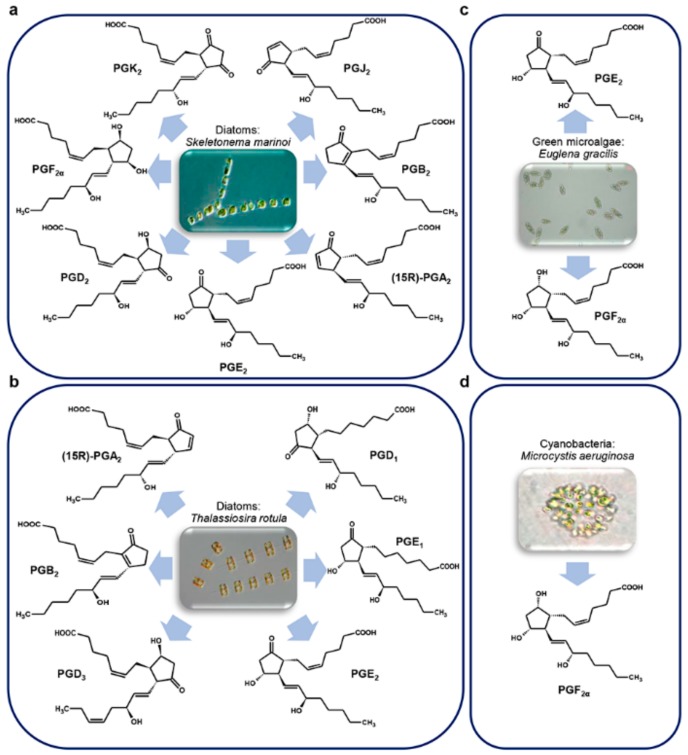
Microalgae prostaglandins. (**a**) *Skeletonema marinoi* (A. Ianora laboratory clones name FE7 and FE60); (**b**) *Thalassiosira rotula;* (**c**) *Euglena gracilis*; (**d**) *Microcystis aeruginosa*. For details, refer to the text.

Di Dato et al. [23] explored the presence of PGs in some diatom species, i.e. *Skeletonema marinoi* Sarno & Zingone, 2005 (Figure 8a) (two different strains, FE7 and FE60) and *Thalassiosira rotula* Meunier, 1910 (Figure 8b) (Valeria Di Dato, Roberta Barbarinaldi, Alberto Amato, Federica Di Costanzo, Carolina Fontanarosa, Anna Perna, Angela Amoresano, Francesco Esposito, Adele Cutignano, Adrianna Ianora, Giovanna Romano. Variation in prostaglandin metabolism during growth of the diatom *Thalassiosira* rotula. *Sci Rep*, under review), identifying in their transcriptome genes involved in PGs biosynthesis, with some differences between the two species. 

More specifically, whereas cyclooxygenase-1 (COX-1) and microsomal prostaglandin E synthase 1 (mPTGES) were found in both species; prostaglandin H_2_ D-isomerase (PTGDS) was found only in *S. marinoi*, while prostaglandin F synthase (PTGFS) was only detectable in *T. rotula*. The authors demonstrated the functioning of the pathway by measuring enzyme expression and molecule production by quantitative real time PCR (qPCR) and liquid chromatography/mass spectrometry (LC/MS) analysis, respectively. Interestingly, a wide set of PGs was revealed with representative molecules of each of the three series. In addition, a differential expression and concentration of molecules was reported among different species and strains of the same species, during different phases of growth and nutrient conditions [23]. However, the absence of PTGDS in *T. rotula* and of PTGFS in *S. marinoi* could be considered as a “potential absence.” Indeed, the lack of a transcript in a transcriptome annotation can be due to a technical shortcoming and a limitation of the sequencing technology used, as in the case of very low expression levels of a target gene, that may fall under the detection limit.

It is interesting to note that, although the PGs pathway has been experimentally confirmed only in two diatom species, in silico analysis of transcriptomes from different species in different growth conditions, suggests the presence of PGs in many other diatom species [23].

Prostaglandins were identified also in the unicellular green alga *Euglena gracilis* G. A. Klebs, 1883 (Figure 8c), in which PGE_2_ and PGF_2α_ were found at levels three times higher in cells grown in the dark than those grown in the light [88].

The presence of PGA, PGE, and PGF series and their esters have also been documented in the cyanobacteria of the taxa *Oscillatoria* and *Microcystidaceae* [89]. Indeed, PGF_2α_ was identified in *Microcystis aeruginosa* (Kützing) Kützing, 1846 [90] (Figure 8d).

Currently, to the best of our knowledge, no other marine microalga has been explored for the presence of PGs, and their role in diatoms is still under investigation. Di Dato and co-authors hypothesized that they can be used by diatoms for intercellular signaling and communication [23], in agreement with the role of these molecules in other organisms.

Microorganisms, and in particular microalgae such as diatoms, could represent a potential source of bioactive PGs, as alternative to their chemical synthesis to produce adequate amounts for pharmacological purposes as anti-inflammatory compounds. The biotechnological production of PGs from microalgae may also be convenient due to the natural presence of the full PGs pathway and the possibility to easily grow microalgae on a large scale in bioreactors under controlled culture conditions.

## 4. Marine Cyclopentenone Prostaglandins

Cyclopentenone prostaglandins are a sub-group of PGs characterized by the presence of a cyclopentenone moiety (CP) with a α,β-unsaturated ketone group in their cyclopentane ring. Their chemical reactivity is responsible for their anti-inflammatory, anti-tumoral and anti-viral activities [91], and the presence of the CP fragment in molecules with anticancer activity, can add more potency to these molecules. For instance, methyl-jasmonate increases its strength of action as an anti-tumor agent when a CP group is inserted in the molecule [92].

The group of cyclopentenone prostaglandins (CPPGs) includes canonical PGs of the A and J series, clavulones, and punaglandins.

Differently from the conventional PGs, that require receptor interaction to trigger their signal, cyclopentenone prostaglandins are actively transported inside the cells [91], where the CP group can interact with a wide variety of target molecules [92] including nuclear factors such as the heat shock protein 70 (HSP70) transcription factor, cyclin-dependent protein kinase (CDK), and NF-ĸB [35] and still not well defined mitochondrial factors [92].

### 4.1. Clavulones and Related Molecules

**Figure 9:**
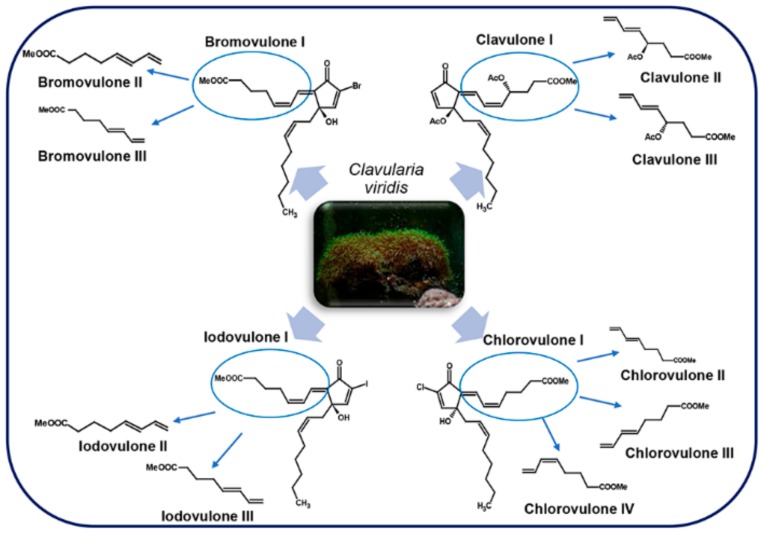
Clavulones and related molecules in *Clavularia viridis* (Cnidaria, soft corals). For details, refer to the text.

Clavulones (Figure 9) are acetoxy derivatives of PGA [91] characterized by a cyclopentenone fragment, a 12-S acetoxy function and two chains of different length: the α- and the ω-chain with respectively seven and eight carbon atoms [93]. These are anti-tumor marine prostanoids isolated from the soft coral *Clavularia viridis* Quoy & Gaimard, 1833, a single *Clavularia* genus that inhabits Okinawa bay in Japan [44,94,95].

Clavulones exists in three isoforms, I, II, and III and two stereoisomers at the C-4 and C-12 positions. These molecules possess a significant anti-inflammatory effect at a concentration of 30 µg/mL in a fertilized chicken egg assay (Table 1) [44,95]. The effects of clavulones on the growth of human cancer cells were first studied using clavulone I, the form that is more abundant in *C. viridis*. At concentration of 4.0 µM there was an impairment of DNA synthesis in human HL-60 leukemic cells after 1 h incubation, causing irreversible cytotoxic changes after 3 h of exposition [45]. In a more recent study, the anti-cancer activity of clavulone II on HL-60 cells has been deeply studied [46] revealing that low concentrations of clavulone II induce anti-proliferative effects by inducing the down-regulation of cyclin D1 with consequent arrest of the cell cycle in G1. On the contrary, higher concentrations of clavulone II induces apoptosis through the disruption of mitochondrial membrane potential and the activation of caspase-9, -8, and -3 and of B-cell lymphoma 2 (Bcl2)-family proteins (Table 1) [46]. It was suggested that corals might use clavulones as repellent and toxic substances against other marine organisms (Table 2) [45].

Clavulones, similarly to PGAs, also possess antiviral activity. In particular, clavulone II inhibits replication of the vesicular stomatitis virus (VSV) in infected mouse L929 fibroblasts, by blocking the transcription of viral RNA, and consequently the viral protein (Table 1) [47].

Interestingly, both the anti-proliferative and antiviral activities of clavulones are stronger than those of PGAs [47].

Other than clavulone I, II, and III, from the same stolonifer coral, 20-acetoxi-claviridenone b and 20-acetoxi-claviridenone c were discovered [96]. In addition to these, also four halogenated analogues, called chlorovulones I-IV were identified and, among these, chlorovulone I was shown to have anti-proliferative and cytotoxic activities (Table 1) [48]. The structure of an epoxy prostanoid with anti-proliferative activity was also identified in the same soft coral [49] together with bromovulone I and iodovulone I. Both had anti-proliferative and cytotoxic activities even though slightly lower than those of chlorovulone I [50]. All these molecules showed a stronger anti-tumor activity against HL-60 cells in vitro with respect to clavulone I (Table 1) [48,49,50].

More recently, seven new prostanoids were identified from the same soft coral: 4-deacetoxyl-12-O-deacetylclavulone I, 4-deacetoxyl-12-O-deacetylclavulone II, bromovulone II, iodovulone II, 4-deacetoxyl-12-O-deacetylclavulone III, bromovulone III, and iodovulone III. These molecules showed in vitro cytotoxic activity against human prostate carcinoma (PC-3) and colon adenocarcinoma (HT-29) cells and, among these, bromovulone III showed the highest anti-tumor activity, together with chlorovulone II and III, used as standard, that exhibited a slightly lower activity (Table 1) [51].

Through an assay-guided fractionation of a dichloromethane (CH_2_Cl_2_) extract of *C. viridis*, three new cytotoxic molecules were isolated: claviridenone E, claviridenone F, and claviridenone G. In particular, claviridenone F showed a significant cytotoxicity against human lung adenocarcinoma (A549), HT-29, and mouse lymphocytic leukemia cells (P-388), while claviridenone G exhibited a high cytotoxic activity only against A549 (Table 1) [52]. In addition to these molecules, there are clavulone-related oxylipins from *C. viridis*, named clavirins I and II, for which it was proposed a derivation from clavulone III and I, respectively, having growth-inhibitory activity against human cervix carcinoma cell line (HeLa S3) (Table 1) [53].

Other clavulone-related molecules that have been discovered are tricycloclavulone and clavubicyclone that may be derived from cycloaddition and electrocyclization of clavulone III, respectively. The latter molecule showed a moderate growth-inhibition activity against breast carcinoma (MCF-7) and ovarian carcinoma cells (OVCAR-3) in vitro (Table 1) [54]. Preclavulone lactone I and II and two minor chemical congeners, 17,18-dehydroclavulone I and clavulolactone I, were also isolated and characterized from *C. viridis*. Among these, 17,18-dehydroclavulone I has a (14Z,17Z)-double bond in the ω side chain, and this suggests that, differently from the other clavulones, it can derive from EPA instead of ARA [97].

Although there are structural similarities between prostaglandins and clavulones, the latter are not generated by a variant of the endoperoxide pathway, but from the lipoxygenase pathway by which arachidonic acid is converted into 8-(R)HPETE and then to preclavulone A [98]. Preclavulone A was identified also in an unrelated Caribbean coral, *Pseudoplexaura porosa* Houttuyn, 1772, suggesting that this molecule is widespread in corals [99]. In another study, the same authors isolated and defined the structures of 15 new halogenated iodo-, bromo-, and chlorovulones in *C. viridis* as minor constituents [100], but overall about 50 congeners of clavulones were identified in this soft coral [101]. 

### 4.2. Punaglandins

**Figure 10:**
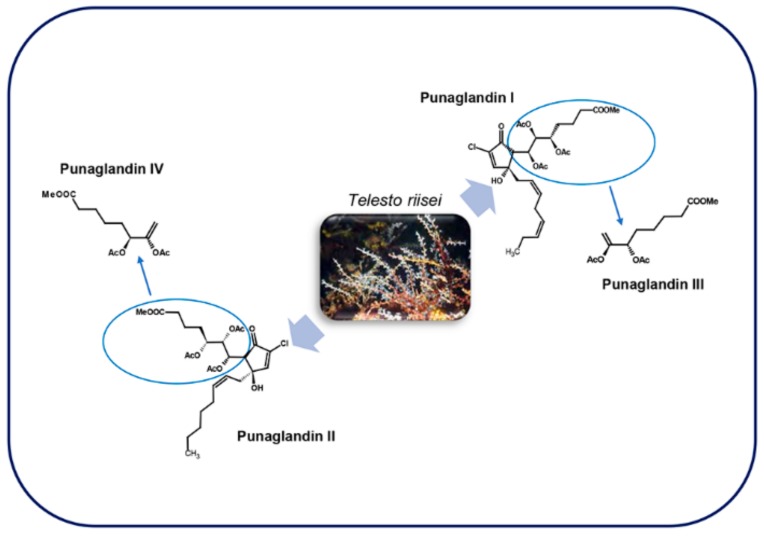
Punaglandins in *Telesto riisei* (Cnidaria, octocoral). For details, refer to the text.

These molecules (Figure 10), structurally related to clavulones, were isolated in the Hawaiian octocoral *Telesto riisei* Duchassaing & Michelotti, 1860 [102]. 

The lack of symbiotic algae suggests that *T. riisei* is the source of these prostaglandins [103]. A total of 19 punaglandins were obtained from this octocoral, which also included acetate and epoxide versions of the four canonical punaglandins [104]. Punaglandins 3 and 4 formally results from elimination of acetic acid from punaglandins 1 and 2 respectively, and possess a cross-conjugated dienone structure [105]. They are halogenated derivatives of PGA [91] characterized by various oxygenations at C -5, -6, -7, and -12 and a 10-chloro-9-cyclopentenone function with a 5S, 6S, 12R stereochemistry [102].

At the moment of their discovery, there was great interest in the study of punaglandins, because of their anti-inflammatory and potent antitumor activities [104]. Indeed, these molecules, and in particular punaglandin 3, was shown to inhibit L1210 leukemia cell proliferation (IC_50_ = 0.04 µM) (Table 1) [102] with an activity 15-fold higher compared to the corresponding clavulone [55]. This cytotoxic activity is approximately equal to the one of vincristine and doxorubicin that are among the most effective anticancer molecules used today [103].

## 5. Marine Thromboxane

Thromboxanes (TXs) are closely relate to PGs that bind specific receptors, called thromboxane receptors (TP) and act as strong promoters of platelet aggregation and vaso- and broncho-constriction in mammals [106]. They are labile and biologically active molecules deriving from arachidonic acid in the cyclooxygenase pathway in human platelets. Thromboxane A_2_ (TXA_2_) has a short half-life (30 s in human blood) being rapidly converted to a stable B form (TXB_2_), which is used as a marker for the presence of TXA_2_ [69].

Other than in mammals, the presence of TXs is documented also in marine organisms.

TXB_2_, along with prostaglandins, has been reported in aqueous homogenates of the mollusc *A. californica* J. G. Cooper, 1863 (Figure 4a) [74] using radioimmunoassay. In the marine bivalve *M. edulis* Linnaeus, 1758 (Figure 4a), arachidonic acid metabolism, in addition to PGs, leads to the production of TXs in the gills, mantle, and adductor tissues [107]. In the polychaetae *Arenicola marina* Linnaeus, 1758 small amounts of TXB_2_ are detectable in the digestive and reproductive tracts [107].

Different species of fish also produce TXs [69]. In the plasma of the elasmobranch *Dasyatis sabina* Lesueur, 1824 (Figure 5b), the presence of an immunoreactive TXB_2_ (iTXB_2_) has been detected at concentrations of 0.57 ± 0.03 ng/mL. The level of iTXB_2_ increased to 3.0 ± 0.27 ng/mL when the plasma clots, but decreased to 1.5 ± 0.17 ng/mL in the presence of indomethacin, a cyclooxygenase inhibitor. These findings suggest that TXA_2_, the active thromboxane form, may be involved in blood clotting and may be generated by a cyclooxygenase-like enzyme as in mammals (Table 2) [69]. The binding of agonist (radiolabeled [125I]-BOP) and antagonist (L-657925 and L-657926) ligands of mammalian TP receptors to those of *D. sabina*, also demonstrates the similarity between this elasmobranch and mammalian TP receptors [69]. Three different species of shark (Figure 5b) (*Chiloscyllium griseum* Müller & Henle, 1838, *Carcharhinus plumbeus* Nardo, 1827 and *Carcharhinus melanopterus* Quoy & Gaimard, 1824) were also shown to produce TXB_2_ as a major product of arachidonic acid metabolism suggesting that TXB_2_ may be a biologically active prostanoid in these species [70].

Rowley et al. [78] tested the ability of leucocytes extracted from the blood of the dogfish *Scyliorhinus canicula* (Figure 5b) to produce and release PGs and TXs during their degranulation induced by the calcium ionophore A23187. While PGF_2α_, PGE_2_, and PGD_2_ levels were always high after the stimulus, the levels of TXB_2_ were low and detectable in the supernatant only 5 min after the start of the stimulus, with levels increasing after 10 and 15 min [78].

Another direct evidence of the generation of TXB_2_ in fish was demonstrated by the ability of washed whole blood cells from a variety of fishes of the Arabian Gulf to produce prostanoids from exogenous 14C-arachidonic acid in vitro [70]. In addition, rainbow trout thrombocytes, clotting cells similar to mammalian platelets, were shown to convert arachidonic acid mainly to TXB_2_ [108,109]. These findings again suggest a role also for TXB_2_, in rainbow trout as a vasodilator (Table 2), while in mammals TXB_2_ has no significant biological activity [70]. However, not all fish thrombocytes have the ability to produce thromboxane, possibly suggesting that these molecules are not essential for thrombocyte aggregation in certain species [110].

## 6. Conclusions

Prostaglandins and their derivatives, although first identified in terrestrial organisms, are also widespread in the marine environment testifying the great importance of these molecules. Nevertheless, the eco-physiological role of marine PGs remains mostly unclear because of contrasting and often old data available in the literature, although it has been suggested that they have a possible role as defensive molecules against predators. 

Interestingly, many marine organisms have been shown to produce classical PGs along with new derivatives that are not found in terrestrial animals, e.g., corals that are able to produce punaglandins and clavulones, which are specific of the marine environment and have shown potent pharmacological activity against inflammation, tumors, and viruses. These findings, together with the discovery of PGs in unicellular eukaryotic microalgae, provides new stimuli to pursue the search for new marine PG-derived molecules with anti-inflammatory activity.

## Figures and Tables

**Table 1:** List of the tested effects of marine prostaglandins, and their derivatives, on mammalian cells.

Prostaglandin	Producer Organism	Activity	Target Cells	Reference
(15R)-PGE_2_ (15R)-*O*-AcPGA_2_	*Plexaura homomalla*	Anti-inflammatory	Leucocyte/TPA-induced mouse-ear edema	Reina et al., 2013 [43]
Clavulones I-III	*Clavularia viridis*	Anti-inflammatory	fertilized chicken eggs	Kikuchi et al., 1983 [44]
Clavulones I-III	*Clavularia viridis*	Anti-cancer	HL-60	Honda et al., 1985 [45]; Huang et al., 2005 [46]
Clavulone II	*Clavularia viridis*	Anti-viral	VSV infected L929	Bader et al., 1991 [47]
Chlorovulone I	*Clavularia viridis*	Anti-proliferative and cytotoxic	HL-60	Iguchi et al., 1985 [48]
PGs Epoxy-prostanoid	*Clavularia viridis*	Anti-proliferative	HL-60	Iguchi et al., 1987 [49]
Bromovulone I and Iodovulone I	*Clavularia viridis*	Anti-proliferative and cytotoxic	HL-60	Iguchi et al., 1986 [50]
Bromovulone III	*Clavularia viridis*	Cytotoxic	PC-3/HT-29	Shen et al., 2004 [51]
Chlorovulones II and III	*Clavularia viridis*	Cytotoxic	PC-3/HT-29	Shen et al., 2004 [51]
Claviridenone F	*Clavularia viridis*	Cytotoxic	A549/HT-29/P-388	Duh et al., 2002 [52]
Claviridenone G	*Clavularia viridis*	Cytotoxic	A549	Duh et al., 2002 [52]
Clavirins I-II	*Clavularia viridis*	Growth-inhibition	HeLa S3	Iwashima et al., 1999 [53]
Clavubicyclone	*Clavularia viridis*	Growth-inhibition	MCF-7/OVCAR-3	Iwashima et al., 2002 [54]
Punaglandins I–IV	*Telesto riisei*	Cytotoxic	L1210	Baker et al., 1985 [55]

**Table 2:** Update of prostaglandins and its derivatives, and functional roles in marine organisms.

Compound	Producer Organism	Biological Activities	Reference
PGF2α	*Marsupenaeus japonicus*	Ovarian maturation	Tahara et al., 2004 [64]
	*Thunnus thynnus*	Contraction of smooth muscles during ejaculation and metabolism of testis	Nomura et al., 1973 [65]
PGE1	Marine Invertebrates	Thermoregulation and fever	Stanley-Samuelson, 1987 [63]
	*Laminaria digitata*	Protection against stress conditions induced by copper excess	Ritter et al., 2008 [19]
	*Salmo sp.*	Contraction of smooth muscles during ejaculation and metabolism of testis	Chirst and Van Dorp, 1972 [60]
PGE2	*Marsupenaeus japonicus*	Ovarian maturation	Tahara et al., 2004 [64]
	*Paralichthys olivaceus* and *Thunnus thynnus*	Contraction of smooth muscles during ejaculation and metabolism of testis	Nomura et al., 1973 [65]
	*Gracilaria vermiculophylla*	Wounding-activated chemical defense molecules	Nylund et al., 2011 [66]
	*Laminaria digitata*	Protection against stress conditions induced by copper excess	Ritter et al., 2008 [19]
PGF2α- and PGF3α-1,15-lactones fatty acid esters (PLFE)	*Tethys fimbria*	Reproduction and multiple roles depending on body localization	Cimino et al., 1991 [67]; Di Marzo et al., 1991 [37]
PGF1α	*Oncorhynchus keta*	Contraction of smooth muscles during ejaculation and metabolism of testis	Nomura et al., 1973 [65]
15-keto-PGE2	*Gracilaria vermiculophylla*	Wounding-activated chemical defense molecules	Nylund et al., 2011 [66]
	*Laminaria digitata*	Protection against stress conditions induced by copper excess	Ritter et al., 2008 [19]
PGE2-1,15-lactone	*Tethys fimbria*	Reproduction and multiple roles depending on body localization	Cimino et al., 1991 [67]; Di Marzo et al., 1991 [37]
PGE3-1,15-lactone-11-acetate	*Tethys fimbria*	Reproduction and multiple roles depending on body localization	Cimino et al., 1991 [67]; Di Marzo et al., 1991 [37]
PGE3-1,15-lactone	*Tethys fimbria*	Reproduction and multiple roles depending on body localization	Cimino et al., 1991 [67]; Di Marzo et al., 1991 [37]
PGD1	*Laminaria digitata*	Protection against stress conditions induced by copper excess	Ritter et al., 2008 [19]
PGA2	*Gracilaria vermiculophylla*	Wounding-activated chemical defense molecules	Nylund et al., 2011 [66]
	*Laminaria digitata*	Protection against copper stress and trigger of oxidative responses	Zambounis et al., 2012 [68]
PGB2	*Laminaria digitata*	Protection against stress conditions induced by copper excess	Ritter et al., 2008 [19]
PGJ2	*Laminaria digitata*	Protection against stress conditions induced by copper excess	Ritter et al., 2008 [19]
Clavulones	*Clavularia viridis*	Suggested to be hypothetical repellents against other marine organisms	Honda et al., 1985 [45]
iTXB2	*Dayatis sabina*	Blood clotting	Cabrera et al., 2003 [69]
TXB2	*Oncorhynchus mykiss*	Vasodilator agent	Thomson et al., 1998 [70]
